# Excited Skin Syndrome (Angry Back), What Do We Know About It? A Review of the Literature

**DOI:** 10.1111/jocd.70568

**Published:** 2026-02-09

**Authors:** Amir Mohammad Beyzaee, Howard I. Maibach

**Affiliations:** ^1^ Department of Dermatology Mazandaran University of Medical Sciences Sari Iran; ^2^ Faculty of Medicine, Department of Dermatology University of California San Francisco (UCSF) San Francisco California USA

**Keywords:** angry back, dermatitis, eczema, excited skin syndrome, patch‐test, skin hyper‐irritability, spill over

## Abstract

**Background:**

In 1975, Mitchell brought up a theory that false positive reactions can be due to skin hyperirritability, called it “angry back syndrome” (ABS). The phenomenon is observed in patch testing of patients with several positive patch tests, mostly along with one or more strong reactions. Later, Maibach rephrased ABS to “excited skin syndrome” (ESS) because of generalized involvement of the skin, not only the back.

**Aims:**

In this paper, we tried to analyze the possible ESS‐related mechanisms and provide crucial information on how to deal with a suspected patient.

**Methods:**

On July 2024, we made a wide systematic computer‐assisted search of *PubMed* and *Google Scholar* (*Embase, Scopus*) data base, using “excited skin syndrome” and “angry back syndrome” keywords. We scanned 350 studies. After removing duplicate studies, 31 studies concerning ESS/ABS were included in our review.

**Results:**

Patch test results with more than a positive reaction can be due to EES development, particularly when the reactions are non‐relevant to the patient. Several factors can alter the chance of ESS including: positioning of the allergens during the patch testing procedure, poly‐sensitization, eczema/dermatitis.

**Conclusions:**

We can consider the following points to manage ESS and minimize its occurrence chance: ESS should be suspected when multiple positive reactions are seen. In suspected cases of ESS, re‐examining and re‐taking history for any relevance of positive reactions is recommended. Retesting should be performed if positive reactions are clinically irrelevant. It is preferred to perform patch testing when no sign of dermatitis is present.

## 1 Introduction

The difficulty of interpreting weak positive patch test reactions, particularly when accompanied by strong positive test reactions, has always been an issue for dermatologists. For the first time in 1975, Mitchell brought up a theory; a hyperirritable skin state may induce false positive reactions, calling it “angry back syndrome” (ABS) [1, 2]. This phenomenon is characterized by observing several positive patch tests along with one (or more) strong (+++) positive reactions in patients administered for epi‐cutaneous battery patch‐testing, in which, positive reactions are not completely reproducible at retesting [3]. Mitchell also added that when a strong positive patch test reaction is observed, other positive reactions are not reliable [4].

In 1981, Maibach rephrased the name of this phenomenon to “excited skin syndrome” (ESS) [3] because he found that it is not restricted to the back and can involve every part of the skin [3, 5].

The exact mechanism of ESS is not fully understood; however, this can be justified by skin hyperirritability resulting from a pre‐existing dermatitis, or fluctuation of humoral and cellular immune response [6].

In this paper, we analyzed the mechanisms and circumstances involved with this phenomenon, and we tried to provide crucial information about ESS and the ways to deal with the suspected patients better.

## 2 Methodology

On July 2024, we made a wide systematic computer‐assisted search of *PubMed* and *Google Scholar* (*Embase*, *Scopus*) data base, using “excited skin syndrome” and “angry back syndrome” keywords. We scanned 350 studies. All types of studies, including case reports, case series, case–control studies, randomized controlled trials, cohort and cross‐sectional studies, animal studies, retrospective and prospective observational studies were reviewed. Furthermore, we checked the references of included studies and articles concerning the ESS/ABS were reviewed; related studies were included. On data acquisition, no language limitation was applied.

After removing duplicate studies, 31 studies concerning ESS/ABS were included in our review.

## 3 Results

Magnusson et al. patch tested adhesive‐tape sensitive patients and compared the results with normal individuals; the difference between the two groups was statistically significant. They concluded that any dermatitis or strong patch‐test reaction can increase skin irritability and cause ESS [7].

In another study, Magnusson stated that during patch testing, positive reaction rate increases when a strong reaction develops [8].

Bruynzeel et al. [9] conducted a retrospective study investigating ESS. Two groups of patients were chosen accordingly: group A was consisted of 157 subjects showing strong positive patch test reactions concomitant with weak positive reactions to a specific allergen. Forty‐five patients whom represented more than one weak positive reaction without any strong reaction were categorized as group B (ICDRG [International Contact Dermatitis Research Group] method was used to score the reactions). All the patients had no sign of active eczema during patch testing. In this study, irrelevant reactions (which were not absolutely near to strong reactions) were reported in 68 patients of group A (43%), and in 13 patients of group B (29%); with no statistical difference between the two groups (*p* > 0.05). Unfortunately, none of the patients were retested and we have no data about the lost reactions. Bruynzeel declared that strong reactions can increase the risk of ESS and because of that, weak reactions are mostly irrelevant [9].

Two years later, Bruynzeel [10] decided to investigate ESS by retesting 61 patients whom represented several positive reactions (strong and weak positive reactions at a same time, or only multiple weak reactions), in a prospective study. After 21 days when the subjects had no sign/symptom of dermatitis, 79 primary weak positive reactions were tested again; of which, 35 (44.3%) were reported to be negative (33 of the 61 patients (54.1%)). They found that lost reactions were much more in patients without dermatitis rather than ones with inactive dermatitis and this difference was significant (*p* < 0.05); The number of lost reactions near to strong positive reactions was higher than distant ones. It was concluded that a hyperirritable skin can be induced by strong reactions, causing several weak positive reactions; weak positive reactions have lack of credit and should be tested again later [10]. Same results were drawn from the studies of Mitchell [1] and Björnberg [11], suggesting chance of false‐positive reaction development is higher near to strong positive reactions.

Maibach et al. [12] tried to quantify ESS patch testing. Fifty‐six patients presenting more than one positive reaction to the basic ICDRG routine series, were retested weekly by one positive reaction‐related agent at a time with identical testing techniques (Al test or Finn chamber on the back for 48 h, with reading at 96 h, utilizing the ICDRG terminology). At retesting, 32 patients (57%) showed reaction to both allergens, 16 (28%) to one allergen, and 8 (14%) had no reaction. Overall, 32 reactions (29%) were lost and ESS was non‐reproducable in 42% of the subjects [12].

Bruynzeel et al. [13] observed that the reaction intensity of sodium lauryl sulphate (SLS) gets significantly lower when test patches are placed distantly from a strong allergic reaction, in 12 of 15 patients (*p* < 0.01). They called it “spillover phenomenon” and stated it could cause false positive reactions [13]; as Fisher had declared that false positive reactions can be caused by spillover phenomenon [14].

Nethercott et al. [15] also studied the irritancy threshold of deodorized kerosene affected by guinea pig maximization test (GMT). 70 guinea pigs were tested accordingly: 10 subjects were patch tested by 3 Al test patches (control group). 20 subjects received four different dosage of intradermal kerosene (0.1 cc) in the shoulder region. Tetra‐ethylene glycol di‐acrylate (TEGDA) was injected intra‐dermally in the shoulder of 20 subjects as well as N‐6 in 20 others with the following dosage respectively: 1.3, 1.6, 2.4, 6 and 1.7, 3, 9, 27 g/100 g in propylene glycol (4 groups of 5 for each substance). No reaction to deodorized kerosene was seen in the control group. More reactions were seen by increasing the topical challenge dosage (*r* = 0.9050, *p* = 0.01); subjects without previous kerosene exposure (only received TEGDA or N‐6) showed an increase in topical challenge by increasing kerosene concentration (*r* = 0.8670, *p* = 0.01). Significant more reactions were observed in 40 subjects with TEGDA and N‐6 exposure, as well as 20 subjects with kerosene exposure rather than control group (*z* = 3.5045, *p* = 0.004/*z* = 2.6944, *p* = 0.008 respectively) [15].

Sonnex et al. [16] surveyed the Trafuril cream effect on ESS. Trafuril is an anxiolytic sedative hypnotic and anti‐psychotic drug that helps to treat symptoms of anxiety such as fear, tension, irritability, and etc.; with high affinity for serotonin 5‐HT1A and 5‐HT2A receptors, and hyperhidrosis and skin rash as side effects. Trafuril upregulates histamine release causing inflammation to the cutaneous vascular system directly. A notable increment in positive patch test reactions was reported, suggesting skin sensitivity can be raised by Trafuril [16].

In 1988, Hamami et al. [17] designed a study consisted of 3 experiments to find the possible abnormalities of a clinically normal skin that could be involved in ESS.

In the first experiment, they entered eight healthy individuals without any history of prior eczema to investigate the barrier function of the stratum corneum and blood flow in normal skin subsequent to a traumatic stimulant. Four subjects received ultra‐violet radiation (UVR) on both sides of the upper back. Adhesive tape was performed on both sides of the back of the other four subjects. Dermal blood flow and transepidermal water loss (TEWL) were measured at days 2, 3, 7, and 14. Forty‐eight hours after the irradiation/stripping, punch biopsies were taken from the administered areas of the back. They found that erythemogenic doses of UVR irradiation increases epidermal thickness, TEWL, and dermal blood flow, and it was statistically significant [17].

In the 2nd experiment, they aimed to evaluate skin temperature and blood flow of the normal skin far from a chemically evoked site. SLS 10% was applied for 4 h on the forearm of 11 control volunteers. After 24 h, blood flow and skin temperature was measured at 2, 5, and 10‐cm away from the inflamated sites (case group), as well as the similar areas on the other arm (control group). No significant difference in blood flow and skin temperature was seen between the two groups [17].

In the 3rd experiment, they aimed to evaluate skin temperature and blood flow of the normal skin distant from an induced allergic contact dermatitis (ACD) site in six nickel‐sensitive patients. Nickel sulphate 5% was applied on the forearm for a complete day. Skin biopsy was performed on five patients. After 24 h of removing the patches, skin temperature and blood flow were increased significantly at the 2‐cm distant site, compared with the control site. A noticeable inflammatory change and epidermal thickening was observed [17].

Romaguera et al. [18] reported a 13‐year‐old boy complaining from maculo‐erythematous/hyperkeratotic lesions on the feet, with a fluctuating status which got worse in the summer. They hired Spanish Contact Dermatitis and Skin Allergy Research Group (GEIDC) standard patch testing series and special shoe series on the back and thin‐layer rapid use epi‐cutaneous patch test (TRUE test) standard allergens on the thighs, when the symptoms were subsided. The reading was done at day 11. They repeated the same patch testing after 1 month; less positive reactions were seen compared to the last times. They concluded that the patient had experienced ESS, caused by foot dermatitis [18].

Gollhausen et al. [19] reported 40% lost reactions during retesting of 41 patients, whom were patch‐tested sequentially with 1 week interval; Similarly, 40% lost reactions by Ruhnek‐Forsbeck [20] and 46.5% by Frosch [21] were reported during repeating patch test application.

Timmer et al. [22] decided to represent a model to study hyper‐sensitivity of skin (ESS). They exposed ten patients suffering from contact allergy to their respective allergens, and thirteen healthy subjects to SLS 5% for a whole day. Then all the subjects were exposed to SLS 0.3% for 24 h on 3 points of the volar forearm at 2, 5, 10 cm away from the test site (allergic/toxic) with 3 similar sites on the other arm, as control. No significant TEWL difference was observed between the two arms nor the groups. However, a considerable difference between the area close to the allergic/toxic site and its control in both groups was recorded by visual scoring and it was statistically significant (*p* < 0.05) [22].

Duarte et al. [23] studied on the rate of ESS among 1500 patients with hypothetical diagnosis of ACD; of which, 34 had more than two positive reactions during the initial patch testing and were retested later with the related agents. ESS rate among 1500 patients was 2.2%. During retesting of 34 subjects, 175 lost reactions (63.5%) were reported [23]; similar to the reported lost rate by Luderschmidt [24] and Nethercott [25] (60%).

Mitchell et al. [26] published a report of two cases of hand eczema with ESS. First case was a 43 years‐old female patient with hand eczema since 8 years ago. She showed positive reaction to balsam of Peru, nickel, and formaldehyde patch tests; while at retesting only nickel was reactive. The next case was a 52 years‐old man suffering from hand eczema since 1 year prior. He was patch tested and diagnosis of ACD to tire rubber was confirmed. At retesting, 62% of initial positive reactions were lost and found to be irrelevant [26].

In another study, Mitchell [2] reported 42% lost reactions while retesting 35 patients whom had 90 one plus (+) patch test reactions. He suggested that false positive reactions can occur when one or more positive reactions are obtained [2].

In 2000, Cockayne et al. [6] studied on 17 patients with ESS; 7 had atopic eczema and 8 had ACD to marginal irritants. They found that marginal irritants had mostly caused ESS [6].

In 2002, Duarte et al. [3] performed epicutaneous tests on 630 ACD‐suspected subjects; the ones whom developed two (or more) positive reactions were retested to investigate ESS. The tests were performed in an inactive dermatitis status. Testing procedure was done accordingly: the retesting was performed with agents showing a positive response in the initial testing, agents with weak/medium reaction were retested 5 cm apart, subjects with multiple strong positive reactions were retested with the same agents one at a time, all tests were applied only after complete remission of the previous positive reactions. ESS developed in 39 of the 630 patients (6.2%). 40.7% of the positive reactions were lost during retesting. Data analysis revealed a longer period of primary dermatitis in patients developing ESS rather than others. Parabens, fragrance mix, and thimerosal showed more positive reactions [3].

In 2019, Gómez Torrijos et al. [27] reported ESS in a neuralgia case. They prescribed carbamazepine and pregabalin for a patient suffering from neuralgia. After 7 days of receiving the treatment, generalized maculo‐papular rash, edema, and fever appeared and the treatment was discontinued. A skin biopsy was taken and diagnosis of drug rash with eosinophilia and systemic symptoms (DRESS) was confirmed. After a 4‐week recovery, allergy tests with pregabalin (Lyrica) 5% and carbamazepine (Tegretol) 5% in both water and pet were applied on the left arm; resulted in positive reaction with both agents in aqua and pet. Two weeks later, the patient was retested with the same agents, each on separate arms; resulted in positive reaction with only carbamazepine. They concluded that the patient has only been allergic to carbamazepine, and pregabalin false positive reaction was obtained at the first test because of patch test proximity [27].

In 2023, Lee et al. [28] reported a young female individual suffering from atopic dermatitis whom was treated with cyclosporine and dupilumab. They decided to perform patch testing to detect any underlying ACD. ESS was developed during patch testing. They declared that regardless the suppressive effect of dupilumab on some allergic reactions, it can not mask ESS on patch testing [28].

However, there are some controversy reports in the literature.

Bandmann et al. [29] surveyed on ESS among 40 patients with leg dermatitis. The subjects were patch‐tested with ICDRG standard series (when dermatitis was improved); of which, patients with two or more positive reactions were retested. Overall, 151 of 1040 initial patch tests reacted positively (14.5%), but only 13 of 151 (8.6%) positive reactions were lost during retesting [29].

Vignale et al. [30] carried out patch testing on 28 ACD patients; 88 positive reactions were observed. Retesting after 8–12 weeks resulted in 14 lost reactions (15.9%) [30].

Kligman et al. [31] aimed to survey ESS on healthy volunteers whom had been manually sensitized by the maximization procedure, in a 4‐part study. In part one, 8 men with strong reactions to I:50 Rhus oleoresin were recruited. SLS reactions were about the same in all subjects, regardless the presence of a strong reaction; strong allergic reactions did not alter the skin reactivity. In part two, 9 nickel‐sensitive individuals with strong reaction to I:50 Rhus oleoresin were entered. The initial reaction to nickel sulphate was not increased by Rhus patch tests. In the 3rd part, the 6 di‐nitro‐chloro‐benzene sensitized subjects did not show any change in inflammatory nor irritant reactions; no sign of ESS was observed. In 4th part, the reactions were equal regardless the distance from strong reaction; No sign of spillover phenomenon was reported [31].

Andersen et al. [32] tested 72 nickel‐sensitive participants hiring a 10‐step dilution of nickel sulphate (TRUE test and two placebo patches). No sign of ESS was observed; neither a statistically significant spillover effect [32].

Memon et al. [4] studied on 32 nickel‐sensitive participants (11 with prior diagnosis of ESS) and 16 non‐sensitive participants with serial fourfold dilutions of nickel sulphate hexahydrate (NiSO_4_.6H_2_O) and croton oil, to investigate the nature of ESS. The assessments were based on ICDRG criteria. Their results failed to represent a specific pattern of skin hyper‐reactivity concomitant with strong reactions to NiSO_4_.6H_2_O; no generalized nor localized change in skin reactivity was observed. The ESS was not reproduced in this study [4].

Duarte et al. [33] performed patch tests on 630 patients with hypothetical diagnosis of contact dermatitis, utilizing standard series of patch tests and hiring an adequate methodology to decrease the ESS chance. 39 subjects showed at least two irrelevant positive reactions; all the 39 subjects were retested and experienced one or more lost reactions (ESS frequency: 6.2%) [33].

## 4 Discussion

Patch test results with more than a positive reaction can be due to EES development, particularly when the reactions are non‐relevant to the patient [10, 27, 34]. The interpretation of weak positive reactions during patch testing is difficult, in particular along with strong reactions. False positives are very important and can be a serious burden on a patient's life, including an unwarranted job change.

Several factors that can alter the chance of ESS are discussed below:

Positioning of the allergens during the patch testing procedure is one of the most important ones; meaning that placing the substances at a greater distance from each other would decrease ESS [3, 10, 23, 27, 34, 35, 36, 37].

In 2002, Duarte et al. [34] observed that substances with chemical affinity would co‐sensitize and show a tendency to have cross‐reactions. In the next year, Brash et al. [38] reported an elevated frequency of positive reactions during patch testing by agents with chemical affinity. Similar structures lead to the secretion of similar haptens [3, 38], resulting in false positive reactions [3, 35]. They concluded that the positioning of the agents during patch testing must be considered [34, 35]. Altering the positioning of the mentioned agents during patch testing of 450 patients, resulted in an overall decrease of ESS and ESS developed in only poly‐sensitized subjects. It supports the theory of ESS development by the positioning of substances with chemical affinity close together [33, 35].

Moreover, poly‐sensitization is assumed as another probable cause of ESS [3, 23, 33, 35]. Poly‐sensitized patients experienced a lower loss rate of positive reactions during retesting in comparison with mono‐sensitized or bi‐sensitized subjects [23].

Eczema is considered another potential cause of ESS. Patients with eczema have a malfunctional skin barrier along with structural abnormalities of the epidermis. The rise of blood flow, malformed stratum corneum and signs of inflammation are found in eczematous skin (regardless of the clinical appearance) which can result in irritancy threshold decrease (skin hyper‐irritability) and extensive skin reactivity which causes false positive reactions/ESS [4, 7, 10, 11, 15, 17, 33, 39]. Both irritant contact dermatitis (ICD) [6, 26, 40] and ACD [6] can make the skin hyperirritable and decrease the irritancy threshold.

Skin hyper‐irritability can be induced by tiny areas of eczema/dermatitis in the affected area and elsewhere; in fact, the entire skin gets affected (not only restricted to the eczematous region) by circulating cytokines that made the dermatitis site hyperirritable [4, 10, 11, 26, 27, 41]. This condition may last more than 12 weeks after dermatitis healing [4, 10, 11, 42]. Accordingly, the strongest positive reactions and significantly fewer lost reactions are reported in induced irritant dermatitis animal models [41, 43, 44, 45], patients with inactive dermatitis [10] and patients with active eczema [4], compared with healthy subjects; also, the spillover phenomenon has been reported in eczema patients as well [2, 10, 13, 23, 26, 46, 47] but not found in non‐eczematous individuals [26, 29, 31].

It is proven that the older the eczema/dermatitis gets the higher the chance of ESS development [3, 23, 48, 49]. In this regard, weak sensitizers (like medicaments, vehicles and preservatives such as neomycin, lanolin) can easily provoke a positive reaction in patients with chronic dermatitis [29]. A strong positive patch test reaction can make minor dermatitis [10], supporting the hypothesis that ESS can be made by strong positive reactions.

When dermatitis gets healed, most inflammatory cells, including Langerhans cells, lymphocytes and mast cells, will remain in the dermis layer for at least 6–8 weeks and will cause skin reactivity enhancement (role of cellular immunity in ESS) [26, 41, 50]. However, the mentioned cells were not increased in the dermis layer of the uninvolved skin (even very close to an area with a strong patch‐test reaction), indicating the role of humoral immunity in ESS development [13, 41].

Marginal irritant allergens (like rubber chemicals, fragrances, metal salts, formaldehyde, parasubstances, parabens, and wool wax alcohols) are mostly responsible for nonspecific false‐positive reactions [6, 10, 41]; when a marginal irritant allergen is applied on a hyperirritable area, based on the concomitant circumstances (like irritancy degree and chemical affinity of the agent, severity and number of positive reactions, reaction site proximity, any prior dermatitis) a false‐positive reaction can be induced [35, 41].

Another noteworthy theory of ESS formation is stochastic resonance; the neurological effect of contact dermatitis can provoke stochastic resonance leading to up‐regulating patch test reactions. Kruglikov et al. [51] declared that the stochastic resonance of a positive patch test symptom (like itching) can alter processes involved with the cell membrane, including up‐regulating vasodilation, lymphokine secretion, and plasma protein extravasation, which result in a visible reaction of sub‐threshold patch tests; whether the noise gets higher, the ESS would expand more [51]. We believe that stochastic resonance can be a cause of cross sensitization, cosensitization, and additive inflammatory response formation leading to ESS. The systemic behavior of ESS [10, 31, 41] is supported by this theory too.

We can see that many procedural and environmental factors contribute in ESS development and altering each one can make controversy results.

Low rate of lost reactions during retesting in Bandmann [29] study can be due to the dermatitis status of the studied subjects. They studied hospitalized patients suffering from stasis dermatitis; however, outpatients were mostly subjects of other studies. Also, they retested two allergens (or more) with strong and weak reactions at the same time which can decrease the lost reaction rate. A low number of tests can also introduce a bias in drawing conclusions.

How about the patch tests that do not react at the initial testing, but show a positive reaction at retesting?

These reactions, called ‘found reaction’, are false‐negative reactions due to a temporary hyporeactive state of skin, also known as ‘sad back’ [26, 45, 52, 53, 54, 55]. It has been shown that induced irritability/inflammation of the skin in humans [41, 56, 57] and animals [41, 58] samples has a biphasic process, a hypo‐reactive state in the early phase of inflammation and a hyper‐reactive state when irritability/inflammation gets more chronic (after 1–4 days). The hypo‐reactive state can be justified by a temporary lack of circulating inflammatory cells attracted to an acute inflammation site [41, 59]. The hyper‐reactive state can be explained by stimulation of bone marrow and humoral immune system responses [41]. Also it is possible that a patch test may not react fast enough to become visible at initial testing [26].

In conclusion, we can consider the following points to manage ESS and minimize its occurrence chance:

- ESS should be suspected when we observe multiple positive reactions, particularly when provoked by marginal irritants.
- In suspected cases of ESS, re‐examining and re‐taking history for any relevance of positive reactions is recommended. However, it is not easy to find the relevance of all reactions.
- Retesting should be performed if positive reactions are clinically irrelevant; particularly when the substance is ubiquitous or is directly relevant to the patient (like his job or living area).
- It is preferred to perform patch testing when no sign of dermatitis is present at any part of the body, or at least dermatitis is in a quiet phase.
- During retesting, each patch test that reacted positively should be tested again, one at a time, at least 6–8 weeks after the skin got healed completely.
- When a related potential allergen is found in history, it is better to be excluded from the initial testing, and be tested at day 4 or day 7 at a different area (like arms) to reduce the chance of ESS.
- When no concentration is recommended for a patch test, ‘normal’ subjects are the best to be tested as controls.
- Irritation should be kept in mind when we have a strong irritant in patch test series or multiple positive reactions are achieved.
- Negative reaction at initial testing should not be considered as an absolute absence of sensitization; in particular when the positive patch tests are not relevant to the patient examination/history.

## 5 Limitations

Despite our deep searching through online databases, we could only find a few articles concerning ESS, mostly published over a decade ago.

## Author Contributions

A.M.B. and H.I.M. designed the research study. A.M.B. performed the research. A.M.B., and H.I.M. contributed essential reagents or tools. A.M.B., and H.I.M. analyzed the data. A.M.B. wrote the original draft. H.I.M. revised the paper (review and editing). H.I.M. supervised the research.

## Funding

The authors have nothing to report.

## Consent

The authors have nothing to report.

## Conflicts of Interest

The authors declare no conflicts of interest.

## Data Availability

The data that support the findings of this study are available from the corresponding author upon reasonable request.

## References

[1] J. Mitchell , “The Angry Back Syndrome: Eczema Creates Eczema,” Contact Dermatitis 1, no. 4 (1975): 193–194. 1235246 10.1111/j.1600-0536.1975.tb05380.x

[2] J. Mitchell , “Multiple Concomitant Positive Patch Test Reactions,” Contact Dermatitis 3, no. 6 (1977): 315–320. 606486 10.1111/j.1600-0536.1977.tb03695.x

[3] I. Duarte , R. Lazzarini , and R. Bedrikow , “Excited Skin Syndrome: Study of 39 Patients,” American Journal of Contact Dermatitis 13, no. 2 (2002): 59–65. 12022121

[4] A. Memon and P. Friedmann , “Angry Back Syndrome': A Non‐Reproducible Phenomenon,” British Journal of Dermatology 135, no. 6 (1996): 924–930. 8977713 10.1046/j.1365-2133.1996.d01-1096.x

[5] A. K. Björk , M. Bruze , M. Engfeldt , C. Nielsen , and C. Svedman , “The Reactivity of the Back Revisited. A Re There Differences in Reactivity in Different Parts of the Back?,” Contact Dermatitis 76, no. 1 (2017): 19–26. 27593358 10.1111/cod.12657

[6] S. E. Cockayne and D. J. Gawkrodger , “Angry Back Syndrome Is Often due to Marginal Irritants: A Study of 17 Cases Seen Over 4 Years,” Contact Dermatitis 43, no. 5 (2000): 280–282. 11016669 10.1034/j.1600-0536.2000.043005280.x

[7] B. Magnusson and L. Hillgren , “Skin Irritating and Adhesive Characteristics of Some Different Adhesive Tapes,” Acta Dermato‐Venereologica 42 (1962): 463–472.

[8] B. Magnusson , “Patch Test Methods: II. Regional Variations of Patch Test Responses,” Acta Dermato‐Venereologica 45 (1965): 257–261. 4162958

[9] D. Bruynzeel , W. Van Ketel , B. von Blomberg‐vander Flier , and R. Scheper , “The Angry Back Syndrome—A Retrospective Study,” Contact Dermatitis 7, no. 6 (1981): 293–297. 7338028 10.1111/j.1600-0536.1981.tb04083.x

[10] D. P. Bruynzeel , W. G. van Ketel , M. von Blomberg‐van der Flier , and R. J. Scheper , “Angry Back or the Excited Skin Syndrome: A Prospective Study,” Journal of the American Academy of Dermatology 8, no. 3 (1983): 392–397. 6833539 10.1016/s0190-9622(83)70044-7

[11] A. Björnberg , Skin Reactions to Primary Irritants in Patients With Hand Eczema (Oscar Isacsons Tryckeri AB, 1968).

[12] H. Maibach , S. Fregert , B. Magnusson , et al., “Quantification of the Excited Skin Syndrome (The ‘Angry Back’) Retesting One Patch at a Time,” Contact Dermatitis 8, no. 1 (1982): 78. 7067445 10.1111/j.1600-0536.1982.tb04149.x

[13] D. Bruynzeel , C. Nieboer , D. Boorsma , R. Scheper , and W. Van Ketel , “Allergic Reactions, ‘Spillover’ Reactions, and T‐Cell Subsets,” Archives of Dermatological Research 275 (1983): 80–85. 6223603 10.1007/BF00412879

[14] A. Fisher , “Patch Test Alert. The ‘Crazy’, ‘Angry,’ Back Has Become the ‘Excited Skin Syndrome’,” Cutis 30, no. 5 (1982): 599–612. 7172740

[15] J. R. Nethercott and M. Lawrence , “The Effect of the Guinea Pig Maximization Protocol on the Irritant Response to Deodorized Kerosene—The Excited Skin Syndrome,” Contact Dermatitis 9, no. 6 (1983): 439–443. 6653100 10.1111/j.1600-0536.1983.tb04459.x

[16] T. Sonnex and T. Ryan , “An Investigation of the Angry Back Syndrome Using Trafuril,” British Journal of Dermatology 116, no. 3 (1987): 361–370. 3567074 10.1111/j.1365-2133.1987.tb05850.x

[17] I. Hamami and R. Marks , “Abnormalities in Clinically Normal Skin—A Possible Explanation of the ‘Angry Back Syndrome’,” Clinical and Experimental Dermatology 13, no. 5 (1988): 328–333. 3256450 10.1111/j.1365-2230.1988.tb00715.x

[18] C. Romaguera , F. Grimalt , and J. Vilaplana , “Allergic Contact Dermatitis From Shoes: ‘Angry Back’ Without ‘Angry Thigh’,” Contact Dermatitis 21, no. 4 (1989): 267. 10.1111/j.1600-0536.1989.tb03207.x 2598654

[19] R. Gollhausen , B. Przybilla , and J. Ring , “Reproducibility of Patch Tests,” Journal of the American Academy of Dermatology 21, no. 6 (1989): 1196–1202. 2584455 10.1016/s0190-9622(89)70329-7

[20] M. Ruhnek‐Forsbeck , T. Fischer , B. Meding , et al., “Comparative Multi‐Center Study With TRUE Test and Finn Chamber Patch Test Methods in Eight Swedish Hospitals,” Acta Dermato‐Venereologica 68, no. 2 (1988): 123–128. 2453990

[21] P. J. Frosch , B. Pilz , D. Burrows , et al., “Testing With Fragrance Mix: Is the Addition of Sorbitan Sesquioleate to the Constituents Useful?,” Contact Dermatitis 32, no. 5 (1995): 266–272. 7634779 10.1111/j.1600-0536.1995.tb00779.x

[22] C. Timmer and P. Van Der Valk , “A Model to Study Non‐Specific Increase in Sensitivity of Human Skin (Excited Skin Syndrome),” Contact Dermatitis 23, no. 4 (1990): 289.

[23] I. Duarte , F. A. Almeida , and N. G. Proença , “Excited Skin Syndrome,” American Journal of Contact Dermatitis 7, no. 1 (1996): 24–34. 8796738 10.1016/s1046-199x(96)90029-9

[24] C. Luderschmidt , G. Heilgemeir , J. Ring , and G. Burg , “Polyvalent Contact Allergy Versus Angry Back Syndrome‐The Problem of False‐Positive Patch Test Reactions,” Allergologie 5, no. 5 (1982): 262–264.

[25] J. R. Nethercott and D. L. Holness , “The Positive Predictive Value of Patch Tests in the Evaluation of Patients With Suspected Contact Dermatitis,” Immunology and Allergy Clinics of North America 9, no. 3 (1989): 549–554.

[26] J. Mitchell and H. I. Maibach , “Managing the Excited Skin Syndrome: Patch Testing Hyperirritable Skin,” Contact Dermatitis 37, no. 5 (1997): 193–199. 9412745 10.1111/j.1600-0536.1997.tb02434.x

[27] E. Gómez Torrijos , A. M. Extremera Ortega , O. Gonzalez Jimenez , J. B. Joyanes Romo , A. R. Gratacós Gómez , and R. Garcia Rodriguez , “Excited Skin Syndrome (‘Angry Back’ Syndrome) Induced by Proximity of Carbamazepine to Another Drug With Strong Positive Allergic Reaction in Patch Test: A First of Its Kind,” Contact Dermatitis 81, no. 5 (2019): 405–406. 31313830 10.1111/cod.13355

[28] M.‐Y. Lee , Y.‐J. Kim , W.‐J. Lee , S.‐E. Chang , M.‐W. Lee , and C.‐H. Won , “Excited Skin Syndrome After Patch Testing in a Patient With Atopic Dermatitis Treated With Dupilumab,” Korean Journal of Dermatology 61, no. 2 (2023): 140–142.

[29] H.‐J. Bandmann and M. Agathos , “New Results and Some Remarks to the ‘Angry Black Syndrome’,” Contact Dermatitis 7, no. 1 (1981): 23. 7249623 10.1111/j.1600-0536.1981.tb03954.x

[30] R. Vignale , P. Pereira , N. Macedo , and A. Carlevaro , “The Irritable Skin Syndrome (‘Angry Back Syndrome’),” Medicina Cutánea Ibero‐Latino‐Americana 10, no. 5 (1982): 313–316. 6764230

[31] A. Kligman and R. Gollhausen , “The ‘Angry Back’: A New Concept or Old Confusion?,” British Journal of Dermatology 115, no. s31 (1986): 93–100. 2943313 10.1111/j.1365-2133.1986.tb02117.x

[32] K. E. Andersen , C. Liden , J. Hansen , and A. Vøeund , “Dose‐Response Testing With Nickel Sulphate Using the TRUE Test in Nickel‐Sensitive Individuals. Multiple Nickel Sulphate Patch‐Test Reactions Do Not Cause an ‘Angry Back’,” British Journal of Dermatology 129, no. 1 (1993): 50–56. 8369211 10.1111/j.1365-2133.1993.tb03311.x

[33] I. Duarte , R. Lazzarini , and R. Buense , “The Excited Skin Syndrome: The Study Realized in 34 Patients: 34,” American Journal of Contact Dermatitis 12, no. 2 (2001): 130. 12022121

[34] I. Duarte , R. Lazzarini , and R. Buense , “Interference of the Position of Substances in an Epicutaneous Patch Test Battery With the Occurrence of False‐Positive Results,” American Journal of Contact Dermatitis 13, no. 3 (2002): 125–132. 12165931

[35] I. Duarte and R. Lazzarini , “Excited Skin Syndrome Associated With Patch‐Test Application Technique,” Dermatitis 17, no. 3 (2006): 161–162. 16956472 10.2310/6620.2006.05032

[36] E. Grosshans and J. Foussereau , “Complications et Complexités de Lecture des Tests Épicutanés,” Annales de Dermatologie et de Vénéréologie; ISSN 0151‐9638; FRA; DA. 110, no. 2 (1983): 259–268. 6614741

[37] D. A. Basketter , “Chemistry of Contact Allergens and Irritants,” American Journal of Contact Dermatitis 9, no. 2 (1998): 119–124. 9601900

[38] J. Brasch , W. Uter , J. Geier , and A. Schnuch , “Associated Positive Patch Test Reactions to Standard Contact Allergens,” American Journal of Contact Dermatitis 12, no. 4 (2001): 197–202. 11753892 10.1053/ajcd.2001.26669

[39] J. V. Klauder and F. A. Brill, Jr. , “Correlation of Boiling Ranges of Some Petroleum Solvents With Irritant Action on Skin,” Archives of Dermatology and Syphilology 56, no. 2 (1947): 197–215. 20257237 10.1001/archderm.1947.01520080057007

[40] S. S. Roper and H. E. Jones , “An Animal Model for Altering the Irritability Threshold of Normal Skin,” Contact Dermatitis 13, no. 2 (1985): 91–97. 4064654 10.1111/j.1600-0536.1985.tb02511.x

[41] D. P. Bruynzeel and H. I. Maibach , “Excited Skin Syndrome (Angry Back),” Archives of Dermatology 122, no. 3 (1986): 323–328. 2937367

[42] M. Grolnick , “Studies in Contact Dermatitis; the Response of Healed Specific Dermatitis Sites to Stimulation With Another Contactant,” Acta Allergologica 1, no. 2 (1948): 229. 18934223

[43] L. van der Lugt and T. S. Veninga , “Nonspecific Hypersensitivity of the Skin,” Dermatologica 162, no. 6 (1981): 438–443. 7274505 10.1159/000250314

[44] K. E. Andersen and H. I. Maibach , “Cumulative Irritancy in the Guinea Pig From Low Grade Irritant Vehicles and the Angry Skin Syndrome,” Contact Dermatitis 6, no. 6 (1980): 430–434. 7438742 10.1111/j.1600-0536.1980.tb04988.x

[45] D. P. Bruynzeel , M. von Blomberg‐van der Flier , W. G. van Ketel , and R. J. Scheper , “Depression or Enhancement of Skin Reactivity by Inflammatory Processes in the Guinea Pig,” International Archives of Allergy and Applied Immunology 72, no. 1 (1983): 67–70. 6874098 10.1159/000234842

[46] H. Maibach , “The ESS—Excited Skin Syndrome (Alias the ‘Angry Back’),” in New Trends in Allergy (Springer, 1981), 208–221.

[47] J. Brasch , T. Henseler , W. Aberer , et al., “Reproducibility of Patch Tests: A Multicenter Study of Synchronous Left‐Versus Right‐Sided Patch Tests by the German Contact Dermatitis Research Group,” Journal of the American Academy of Dermatology 31, no. 4 (1994): 584–591. 8089284 10.1016/s0190-9622(94)70220-9

[48] I. A. G. Duarte and F. A. D. Almeida , Sindrome da pele excitada (Angry Back) (Universidade de São Paulo, São Paulo, 1994).

[49] M. Hindsén , M. Bruze , and O. B. Christensen , “The Significance of Previous Allergic Contact Dermatitis for Elicitation of Delayed Hypersensitivity to Nickel,” Contact Dermatitis 37, no. 3 (1997): 101–106. 9330814 10.1111/j.1600-0536.1997.tb00312.x

[50] S. Rj , “Induction of Immunological Memory in the Skin: Role of Local T‐Cell Retention,” Clinical and Experimental Immunology 51 (1983): 141–148. 6600990 PMC1536743

[51] R. L. Rietschel , “Stochastic Resonance and Angry Back Syndrome: Noisy Skin,” American Journal of Contact Dermatitis 7, no. 3 (1996): 152–154. 8957329

[52] D. Bruynzeel and H. Maibach , “Excited Skin Syndrome and the Hyporeactive State: Current Status,” in Exogenous Dermatoses: Environmental dermatitis (CRC Press, 1990), 141–150.

[53] D. Todd , J. Hasdlev , M. Metwali , G. Allen , and D. Burrows , “Day 4 Is Better Than Day 3 for a Single Patch Test Reading,” Contact Dermatitis 34, no. 6 (1996): 402–404. 8879925 10.1111/j.1600-0536.1996.tb02241.x

[54] K. Lammintausta , H. Maibahi , and D. Wilson , “Human Cutaneous Irritation: Induced Hyporeactivity,” Contact Dermatitis 17, no. 4 (1987): 193–198. 2962817 10.1111/j.1600-0536.1987.tb02712.x

[55] M. Uehara and S. Ofuji , “Suppressed Cell‐Mediated Immunity Associated With Eczematous Inflammation,” Acta Dermato‐Venereologica 57, no. 2 (1977): 137–139. 71808

[56] H. Hering , “Zur Beurteilung berufsbedingter Hauterkrankungen unter Berücksichtigung von Fehlerquellen bei epikutanen Testproben,” Dermatologische Wochenschrift 130 (1954): 1129. 13231539

[57] A. M. Kligman , “Poison Ivy (Rhus) Dermatitis: An Experimental Study,” AMA Archives of Dermatology 77, no. 2 (1958): 149–180. 13497290 10.1001/archderm.1958.01560020001001

[58] J. Frey , “Quantitative Untersuchungen bei epidermaler Sensibilisierung von Meerschweinchen mit Dinitrochlorbenzol,” Dermatology 102, no. 1 (1951): 1–11. 14822622

[59] R. L. McGuire and R. Fox , “Suppression of Delayed Hypersensitivity by the Depletion of Circulating Monocytes,” Immunology 38, no. 1 (1979): 157–161. 511215 PMC1457912

